# Supplementary material for: KMT2D/MLL2 inactivation is associated with recurrence in adult-type granulosa cell tumors of the ovary
Source: Nat Commun. 2018 Jun 27;9:2496. doi: 10.1038/s41467-018-04950-x (PMC6021426; doi:10.1038/s41467-018-04950-x)
Supplement: Supplementary file 2 — Description of Additional Supplementary Files [file 41467_2018_4950_MOESM2_ESM.pdf]

Description of Additional Supplementary Files

File Name: Supplementary Data 1
Description: Clinical summary of adult type ovarian granulosa cell tumor frozen tissue discovery cohort.

File Name: Supplementary Data 2
Description: Sequencing metrics for whole exome sequencing of adult type ovarian granulosa cell tumor frozen tissue discovery cohort.

File Name: Supplementary Data 3
Description: Summary of coding mutations identified in adult type ovarian granulosa cell tumor frozen tissue discovery cohort.

File Name: Supplementary Data 4
Description: TruSeq Custom Amplicon regions used for deep amplicon sequencing of FOXL2 and KMT2D coding regions.

File Name: Supplementary Data 5
Description: Sequencing metrics for cancer gene panel sequencing of adult type ovarian granulosa cell tumor FFPE validation cohort.

File Name: Supplementary Data 6
Description: Automated image analysis summary for KMT2D immunohistochemistry and automated image analysis.
